# Consumer Acceptance of Meat from Animals Reared on Insect Meal as Feed

**DOI:** 10.3390/ani10081312

**Published:** 2020-07-30

**Authors:** Katalin Szendrő, Mónika Zita Nagy, Katalin Tóth

**Affiliations:** 1 Institute of Marketing and Management, Kaposvár University, H-7400 Kaposvár, Hungary; 2 Institute of Methodology, Kaposvár University, H-7400 Kaposvár, Hungary; nagy.monika@ke.hu; 3 Institute of Regional and Agricultural Economics, Kaposvár University, H-7400 Kaposvár, Hungary; toth.katalin@ke.hu

**Keywords:** insect, farmed animals, meat, consumer, acceptance

## Abstract

**Simple Summary:**

Poultry, pigs, and fish consume insects in nature and therefore insect meal could be considered to be an acceptable substitute for soybean and fishmeal as a protein source in the diets of these animals. Hungarian respondents were asked about their opinion on consuming meat obtained from animals under free-range conditions or from animals that had received insect meal in their diet. On a scale of 1–7, the respondents gave 5.11 points for the meat of free-range animals, but significantly less points (3.69) for the meat of animals that had consumed insect meal. However, free-ranging animals also consume insects and other small animals, of which the origin is not known, while the insects used as feed are produced under controlled conditions. The consumers’ decisions are affected by their attitudes: free-range is associated with animal welfare, but mentioning insects creates feelings of aversion and antipathies. In conclusion, to increase the consumers’ acceptance of meat products from animals reared on insect meal, consumers need to be made aware of the various benefits of insect meal in animal feed.

**Abstract:**

Insect protein production requires much less land, feed, and water, and thus has a much smaller ecological footprint than animal protein production, which is important for reducing global warming. Poultry, pigs, and fish consume insects in nature, so insect meal could be a good substitute for soybean and fishmeal as a protein source in diets. The aim of this study was to examine consumer opinion on meat that originated from animals whose diet contained insect meal. The study was conducted in Hungary in 2020 (*N* = 414). On a scale of 1–7, respondents gave much lower scores (3.96) to this product than that which originated from a free-range system (5.11; *p* < 0.001). Male, more highly educated, and 30–39 year-old respondents gave significantly higher scores than other groups. The most important factor in accepting insect meal in animal feed was “no risk”, while the least important factor was “replacement of Genetically Modified (GM) soybeans”. Since free-range animals also consume insects, the difference in the attitude of respondents was based on their aversion to insects, while accepting free-range as the best animal welfare system. Thus, more emphasis should be placed on the benefits of insect meal in animal feed in order to bring about awareness and acceptance.

## 1. Introduction

People have been eating insects (adults and their eggs, larvae, and pupae) since ancient times [1,2]. They are a very important source of protein and have other high nutritional values [3,4]. Today, they are still consumed in several African, Asian, and South American countries [5]. Very few Europeans eat insects; the majority are unfamiliar with the idea, and reject it. Many are also disgusted with the consumption of insects and food containing insects [6]. However, in recent times some Europeans, especially young people, are willing to taste them [7]. This is partly because insect protein production requires much less land, feed, and water, and thus has a much smaller ecological footprint than animal protein production [8], which is important for reducing global warming [9]. At the same time, in countries where insect consumption is common, an increasing shift to a Western diet occurs [10,11].

It would have a positive impact on the environment if insect meal played a greater role in the feed of farmed animals as a protein and possible energy source. The omnivorous farmed animals, such as poultry, pigs, and fish, like consuming insects and other animals living in the soil, on its surface, or even in the air. Today, the major sources of protein for these animals are soybean meal and fishmeal. In recent decades, the amount of marine fish production has fallen and the price has increased significantly, and this trend is expected to continue [12]. The price of soybean is also increasing, with some fluctuations [13]. Genetically Modified Organisms (GMOs) can also be a problem in Europe, which is common in the case of imported soy. Insect meal could be a good alternative to fishmeal and soymeal. Generally, insect meal contains the whole insect as a source of protein, energy, and fatty acids (FAs). Moreover, when using defatted insects, they are rich in protein and the insect oil could be used as a good source of omega-3 FAs. The oil composition depends on the species and the feed of the insects [14,15]. In recent years, several papers have been published that show that fishmeal and soy can be partially or completely replaced by insect meal in poultry [16,17,18], in pigs [19], and in fish [20,21]. Despite the positive results, the European Union (EU) only allows insects to be used as a source of protein in fish feed [22]. However, it is expected that the authorization for poultry and pig feed will be completed within a short time.

A sufficient number of insects and amount of insect flour can be produced, in large quantities and of guaranteed quality, only under industrial (farm) conditions. Like with farm animals, animal welfare issues can arise, such as issues with pain, feeling, comfort, and well-being. Although in most cases there is no clear answer to these questions, caution should be exercised, and insect production must be continued with care, with particular attention given to their killing [23]. Several papers have been published in the field on customer opinion about eating insects, or different products that include insects [24,25,26,27,28]. However, only a limited number of studies evaluated the respondents’ opinion about consuming meat or other animal products that originated from animals fed with diets including insect meal [29,30,31]. The aim of the present study was to examine the respondents’ opinion on this type of meal.

## 2. Materials and Methods

The study was conducted in Hungary in 2020. The survey asked the respondents to quantify their opinion, on a 1–7 scale, regarding meat that originated from animals kept in a free-range system or from animals fed with insect meal, and on factors influencing the acceptance of an animal product fed with insect meal.

Among non-probability sampling techniques, snowball sampling of data collection [32] was used, meaning that the structured survey was given to an initial group of respondents (those who used the Internet) selected randomly. Respondents were encouraged to locate other members of the target population whom they knew; e.g., friends, relatives, and colleagues. The total number of responses was 414 (Table 1). Subgroups were formed based on the background information: gender, age, education, residence, and household income.

### Statistical Analysis

Only faultless questionnaires were evaluated. The questionnaire was evaluated with one-way ANOVA using IBM SPSS 22.0 software (IBM, New York, NY, USA):

Y_ij_ = μ + V_i_ + e_ij_ (1)

where: μ = general mean, V_i_ = effect of the variables (i = 1–2), and e_ij_ = random error. Cross tables (for determining the relation of a variable to the background variables and to other involved variables) were used in the evaluation of the questionnaires. The significance of differences was tested by Tukey’s post hoc test. In addition, a *t*-test was performed to verify proven differences, and descriptive statistical methods were used. For background variables, those respondents whose proportion did not reach 3% were excluded from the analyses due to the low number of items.

## 3. Results

Most of the respondents (98%) regularly consumed meat or other protein of animal origin.

### 3.1. Interest in the Feeding of Animals Whose Meat or Other Product Is Consumed

On a 1 to 7 scale, the average score was 4.54. The results show that people are generally not particularly interested in what kind of feed the animals consumed. The given scores were independent of gender, age, residence, and household income. Difference was found at a *p* = 0.088 level only in education (secondary school: 4.25, higher education: 4.63). It can be seen that people generally believed that animals were fed properly, so consumers did not pay much attention to it.

### 3.2. Preference of Choosing Animal Products That Originated from Animals Kept in a Free-Range System

Scores slightly above 5 were mostly given for this question (average: 5.11). The effects of age, residence, and household income were not significant. A highly significant difference was found between genders (female: 5.31, male: 4.72; *p* = 0.003). The more highly educated respondents gave higher scores (5.20) than the secondary school graduates (4.82); however, it was proven only at a *p* = 0.086 level.

Most people tended to associate the free-range system with animal welfare. The consumer perception of free-range livestock farms indicates an idyllic scenario [33] at the top of animal welfare. Consumers have a positive attitude towards more animal-welfare-friendly systems, with outdoor access, and are ready to pay a higher price for products from such production systems [34]. Free-range chicken meat and eggs had the highest relative importance for consumers compared with organic, and especially conventional, products [35,36,37]. This could be the reason why respondents gave high scores for animal products that originated from an animal reared in a free-range system. According to a meta-analysis among the most important factors, consumers prefer systems for pig and poultry that have more space and outdoor access [34]. However, hens, chickens, etc. do not like to stay outside when it is too cold, warm, rainy, or windy. In line with the results of the present study, Maria [38] showed that women and more highly educated people were more sensitive to the conditions under which animals were reared.

### 3.3. Willingness to Consume Animal Products That Originated from Animals Fed with Insect Meal

The use of insect meal for animal feeding purposes is regulated by law. Lahteenmaki-Uutela et al. [39] have analyzed in detail the EU’s and some other countries’ regulations on the use of insects as food and feed. In the EU, it can be used as feed in aquaculture, but it remains banned for other farmed animals. As the ban is expected to be lifted, intensive research has been conducted in several countries.

Respondents gave the lowest score (3.96) for this question with the largest difference between the factors. Males gave higher scores than females (4.22 and 3.41, *p* < 0.001), respondents with higher education gave higher scores than secondary school graduates (3.96 and 2.88, *p* < 0.001), and the effect of age was also significant (Table 2). The highest scores were given by respondents of the 30–39 age group and the lowest ones by the oldest and the youngest groups. The richest group gave the highest scores (Table 3); however, the differences between groups were significant only at the *p* = 0.052 level.

As it is legal to feed fish with insects, practical answers can be obtained; however, as it is not yet allowed in case of other farmed animals, the answers can be considered to be theoretical ones, but important for the future.

A study of consumers’ acceptance of fish fed with diets containing insects was conducted in Italy [40]. Almost 50% of the consumers fully accepted the fish and 40% partially accepted it, while it was rejected by a minority of consumers. In another study in Scotland [41], more than 50% of the respondents accepted eating salmon fed with an insect-derived diet. Another 36% answered that they would be willing to accept the fish if the price, safety, and taste remained unchanged, while 10% were unwilling to eat insect-fed fish. In a survey [42], it was found that 77% of Danish consumers were indifferent to feeding fish with an insect-containing diet, but the remaining 23% were sensitive to it. The acceptance of trout was compared according to their consumption or not of insects [43]. Most of the respondents in France (61%) agreed or strongly agreed that in nature fish eat insects. However, 15% of them said it was disgusting to eat trout that had eaten insects.

The acceptance of meat from insect-fed animals in other countries is generally similar to that found in our study. Belgian citizens were asked about mixing insect meal into feed for farmed animals [29]. The score for attitude using insects in animal feed was 3.89 on a scale of 1–5. The highest values were given if the insects were in feed for poultry and fish, rather than for pigs or cattle. In an Italian study [30], the respondents were divided into three groups: those who in most cases agreed to (A), those who were uncertain about (U), and those who disagreed with (D) mixing insects into animal diets. They found that 53%, 25%, and 22% of participants in the A, U and D groups, respectively, accepted the incorporation of insects into animal diets, and would eat the meat of these livestock. Brazilian authors [31] investigated the willingness to accept the use of insects in feed. The scores for the opinion of the respondents about using insects in the feed of animals were generally near to 3, on a 1–5 scale, with the highest values for fish and the lowest ones for cattle. Surprisingly, the lowest acceptance results have been published in Brazil, where some people still consume insects [44].

Interestingly, consumers more or less refuse to consume meat or fish that were fed with insect meal. At the same time, they would be willing to pay a higher price for products from free-range animals. However, these animals also eat different animal origin feed. Pigs are omnivores, which means that they consume both plants and animals. They primarily eat leaves, roots, and fruit, in addition to some insects and other animals. Chickens, turkeys, and guinea fowl consume a variety of invertebrates, especially arthropods [22,45]. Insects are also part of the natural diets of carnivorous and omnivorous fish [46]. People eat the meat of wild boar, pheasants, fish, and other animals that consume insects of unknown origin without any aversion. At the same time, respondents refrain from consuming meat from farmed animals and fish whose feed was mixed with insects produced under controlled conditions. All this shows that there is a great need to bring about awareness in order to increase the consumption of animal products fed with insect meal without any antipathy.

The t-test verified proven differences among variables of interest in the feeding of animals whose meat or other product is consumed, the preference for choosing animal products that originated from animals kept in a free-range system, as well as the willingness of consuming animal products that originated from animals fed with insect meal.

### 3.4. Factors Influencing the Acceptance of a Product from an Animal Fed with Insect Meal

Factors influencing the acceptance of products of insect-fed animals were examined in more detail.

**Figure 1:**
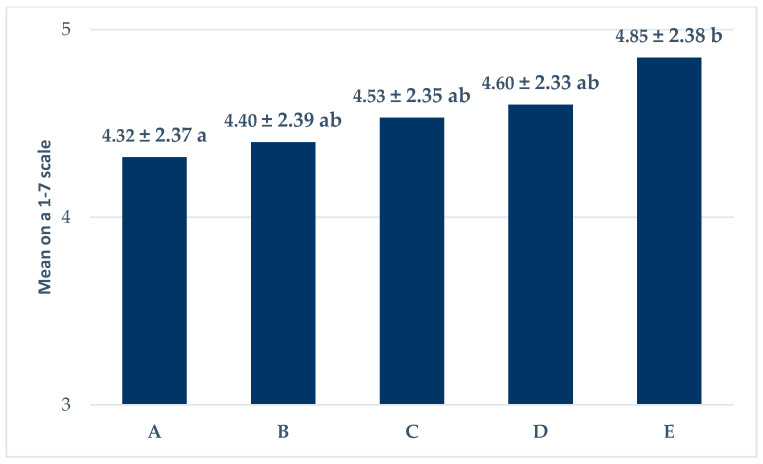
Factors influencing the acceptance of products that originated from animals fed with insect meal (means ± SD; on a 1 to 7 scale; A: replaced GM soybean; B: produced under controlled conditions; C: positive effects on human health; D: smaller ecological footprint; E: no risk. ^a,b^ means where different superscripts differ (*p* < 0.05).

Respondents gave different scores for factors influencing the acceptance of products that originated from animals fed with insect meal (Figure 1). The results show that “no risk” played the most important role in accepting a product that originated from an animal fed with insect meal, while “replacing Genetically Modified (GM) soybean” had the least influence (*p* < 0.05). It seems that consumers are not afraid of animals fed with GM soy meal. Other factors were in between, with no significant differences.

Men gave higher scores for all factors than women by 0.56 points on average (Table 4). The largest difference was found in the case of animals fed with insects that were produced under controlled conditions. Women gave one of the lowest scores, while men gave a fairly high score, for this factor.

The effect of age was significant in each factor (Table 5). Respondents aged 30–39 years gave the highest scores, while the lowest scores were given by the oldest group (>50), and also the youngest, in the case of GM soybean replacement. The highest acceptance was found for products, originated from animals fed with insect meal, that had no risk.

Higher-educated respondents gave significantly higher scores for all factors than those of secondary school graduates by 0.76 points on average (Table 6). The differences were very similar in each factor (0.78–0.85), except for “no risk” (0.60). The effect of education was higher than that of gender.

A statistically significant difference was not apparent with regard to the residence of the respondents. Household income did not affect the given score either.

The choice of Hungarian respondents was least influenced by replacing GM soy with insect meal. GM products are considered very differently around the world. Consumers have both positive and negative perceptions. Known and perceived benefits and risks play a significant role in behavioral intentions towards GM food, contingent on attitudes towards GM technology [47]. People have quite a bit of knowledge about the advantages and disadvantages of GM technologies. Similarly, in the present study, males had more tolerant attitudes towards GM foods than females, and older customers were willing to pay more for non-GM food products [47].

Our results show that several factors influence the acceptance of animals fed with insect meal (they are shown in Figure 1, Table 4, Table 5 and Table 6, and in the text) and are in line with the findings of other authors in many cases. According to Laureati et al. [30], the willingness to accept this kind of meat and fish was higher in males than females, and higher in students and university staff than outside the university. The ratio of preference was also higher in males and younger age groups in the study of Ankamah-Yeboah et al. [42]. Verbeke et al. [29] also observed that feeding animals with insects was more acceptable for men than for women; however, it was independent of age. In another study [44], gender and education level did not affect the willingness to accept using insects in the feed of poultry, pigs, fish, or cattle, while older people accepted this feed for fish less. Popoff et al. [41] did not find any differences among the different social groups. Information on feeding insects influenced the choice. If there was no difference in price, 54.5% of the informed people would prefer the trout fed with insects, while only 39.4% of the non-informed group would [43].

## 4. Conclusions

Insect meal could be a good protein source in diets for poultry, pigs, and fish. Although most Hungarian respondents accepted the meat of insect-fed animals with no reservations, there were, however, still others who rejected it. At the same time, animal products from a free-range system were more positively received, even though these animals also eat insects. This may be because people associate the free-range system with good animal welfare, while some of the respondents may reject insects mixed with feed (or have insect phobia). Thus, more emphasis should be placed on the benefits of insect meal in animal feed in order to achieve greater awareness and acceptance of the benefits of insect-based animal feed.

## Figures and Tables

**Table 1:** The distribution of the sample.

Description	*n*	%
**Total Respondents**	414	100
**Gender**		
Female	271	65.5
Male	143	34.5
**Age**		
18–29	98	23.7
30–39	99	23.9
40–49	100	24.2
>50	117	28.3
**Educational Background**		
Secondary School	101	24.4
College, University	313	75.6
**Residence**		
City	318	76.8
Village	96	23.2
**Household Income**		
Just enough, but cannot set any money aside	63	15.2
Live well but can only set little money aside	205	49.5
Live very well and with high enough income to set money aside	120	29.0
No answer/Do not know	26	6.3

In the questionnaire, it was emphasized that the survey was anonymous, and consent was asked for at the first point to publish the results in a scientific paper.

**Table 2:** Preference for animal products that originated from animals fed with insect meal depending on the age of respondents on a 1–7 scale.

Age, Years	Mean	SEM
18–29	3.41 ^a^	0.21
30–39	4.48 ^b^	0.22
40–49	3.86 ^ab^	0.21
>50	3.08 ^a^	0.20
*p*-Value	<0.001	

^a, b^ means with different superscript in a column differ (*p* < 0.05).

**Table 3:** Preference for animal products that originated from animals fed with insect meal, depending on the household income of respondents, on a 1–7 scale.

Level of Income	Mean	SEM
Just enough, but cannot set any money aside	3.52	0.27
Live well but can only set a little money aside	3.56	0.15
Live very well and with high enough income to set money aside	4.18	0.20
*p*-Value	0.052	

**Table 4:** Factors influencing the acceptance of a product that originated from animals fed with insect meal, depending on gender (on a 1–7 scale).

I Would Choose an Animal Product Fed with Insect Meal if I Knew That …	Female		Male		*p*-Value
	Mean	SEM	Mean	SEM	
GM soybeans were replaced.	4.17	0.15	4.60	0.20	0.089
it was produced under controlled conditions.	4.14	0.15	4.86	0.20	0.004
it had positive effects on human health.	4.30	0.15	4.94	0.19	0.010
it had a smaller ecological footprint.	4.42	0.15	4.91	0.19	0.047
it had no risk.	4.67	0.15	5.17	0.20	0.048

**Table 5:** Factors influencing the acceptance of products that originated from animals fed with insect meal, depending on the age of respondents (on a 1 to 7 scale).

I Would Choose An Animal Product Fed Insect Meal if I Knew that …	Age, Years								*p*-Value
	18–29		30–39		40–49		>50		
	Mean	SEM	Mean	SEM	Mean	SEM	Mean	SEM	
GM soybeans have been replaced.	3.78 ^a^	0.24	5.04 ^b^	0.23	4.43 ^ab^	0.26	4.04 ^a^	0.24	0.002
it was produced under controlled conditions.	4.23 ^ab^	0.24	4.93 ^b^	0.23	4.69 ^ab^	0.24	3.84 ^a^	0.24	0.006
it had positive effects on the human health.	4.39 ^ab^	0.23	5.09 ^b^	0.23	4.75 ^ab^	0.24	4.00 ^a^	0.24	0.006
it had a smaller environmental footprint.	4.22 ^ab^	0.22	5.22 ^c^	0.22	4.97 ^bc^	0.24	4.02 ^a^	0.24	<0.001
it had no risk.	4.75 ^ab^	0.24	5.30 ^b^	0.23	5.21 ^b^	0.24	4.21 ^a^	2.56	0.004

^a–c^ means with a different superscript in a column differ (*p* < 0.05).

**Table 6:** Factors influencing the acceptance of a product that originated from an animal fed with insect meal, depending on educational background (on a 1–7 scale).

I Would Choose an Animal Product Fed Insect Meal if I Knew That …	Secondary School		Higher Education		*p*-Value
	Mean	SEM	Mean	SEM	
GM soybeans were replaced.	3.72	0.25	4.51	0.14	0.006
it was produced under controlled conditions.	3.79	0.25	4.59	0.14	0.005
it had positive effects on human health.	3.94	0.24	4.72	0.14	0.005
it had a smaller environmental footprint.	3.95	0.24	4.80	0.13	0.002
it had no risk.	4.39	0.26	4.99	0.14	0.036
